# *N*-[2-(3,4-Dimeth­oxy­phenyl)eth­yl]-*N*-methyl­naphthalene-1-sulfonamide

**DOI:** 10.1107/S1600536812008203

**Published:** 2012-02-29

**Authors:** Jasmine P. Vennila, D. John Thiruvadigal, Helen P. Kavitha, G. Chakkaravarthi, V. Manivannan

**Affiliations:** a Department of Physics, Panimalar Institute of Technology, Chennai 602 103, India; b Department of Physics, SRM University, Kattankulathur Campus, Chennai, India; c Department of Chemistry, SRM University, Ramapuram Campus, Chennai 600 089, India; d Department of Physics, CPCL Polytechnic College, Chennai 600 068, India; e Department of Research and Development, PRIST University, Vallam, Thanjavur 613 403, Tamil Nadu, India

## Abstract

In the title compound, C_21_H_23_NO_4_S, the dihedral angle between the naphthalene residue and the benzene ring is 7.66 (3)°. In the molecule, there are some short C—H⋯O interactions. In the crystal, the structure is stabilized by weak intra­molecular C—H⋯O hydrogen bonds and the crystal structure is stabilized by weak C—H⋯O, C—H⋯π and π–π [centroid–centroid distance = 3.710 (2) Å] inter­actions.

## Related literature

For biological activities of sulfonamide derivatives, see: Schultz *et al.* (2001 ▶); Sheppard (2006 ▶); Xiong *et al.* (2007 ▶). For related structures, see: Vennila, Thilagavathi *et al.* (2008 ▶); Vennila, Kavitha *et al.* (2008 ▶).
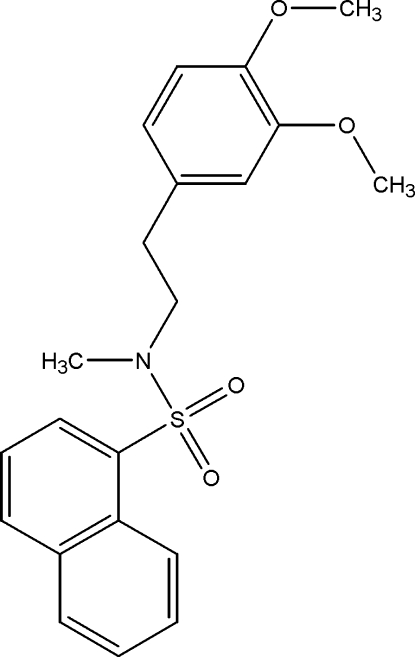


## Experimental

### 

#### Crystal data

C_21_H_23_NO_4_S
*M* *_r_* = 385.46
Orthorhombic, 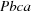

*a* = 10.070 (5) Å
*b* = 14.120 (4) Å
*c* = 27.229 (5) Å
*V* = 3872 (2) Å^3^
*Z* = 8
Mo *K*α radiation
μ = 0.19 mm^−1^
*T* = 295 K
0.34 × 0.30 × 0.28 mm

#### Data collection

Bruker Kappa APEXII diffractometer
Absorption correction: multi-scan (*SADABS*; Sheldrick, 1996 ▶) *T*_min_ = 0.937, *T*_max_ = 0.948
9215 measured reflections
5239 independent reflections
3248 reflections with *I* > 2σ(*I*)
*R*_int_ = 0.041
Standard reflections: 0

#### Refinement

*R*[*F* ^2^ > 2σ(*F* ^2^)] = 0.045
*wR*(*F* ^2^) = 0.120
*S* = 1.01
5239 reflections
247 parameters
H-atom parameters constrained
Δρ_max_ = 0.21 e Å^−3^
Δρ_min_ = −0.38 e Å^−3^

### 

Data collection: *APEX2* (Bruker, 2004 ▶); cell refinement: *SAINT* (Bruker, 2004 ▶); data reduction: *SAINT*; program(s) used to solve structure: *SHELXS97* (Sheldrick, 2008 ▶); program(s) used to refine structure: *SHELXL97* (Sheldrick, 2008 ▶); molecular graphics: *PLATON* (Spek, 2009 ▶); software used to prepare material for publication: *SHELXL97*.

## Supplementary Material

Crystal structure: contains datablock(s) global, I. DOI: 10.1107/S1600536812008203/bt5826sup1.cif

Structure factors: contains datablock(s) I. DOI: 10.1107/S1600536812008203/bt5826Isup2.hkl

Supplementary material file. DOI: 10.1107/S1600536812008203/bt5826Isup3.cml

Additional supplementary materials:  crystallographic information; 3D view; checkCIF report

## Figures and Tables

**Table 1:** Hydrogen-bond geometry (Å, °) *Cg*2 and *Cg*3 are the centroids of the C2–C7 and C14–C19 rings, respectively.

*D*—H⋯*A*	*D*—H	H⋯*A*	*D*⋯*A*	*D*—H⋯*A*
C3—H3⋯O2	0.93	2.53	3.132 (3)	122
C10—H10⋯O1	0.93	2.37	2.804 (3)	109
C11—H11*B*⋯O2	0.96	2.34	2.821 (3)	110
C4—H4⋯O3^i^	0.93	2.46	3.310 (3)	152
C8—H8⋯*Cg*3^ii^	0.93	2.59	3.488 (3)	163
C20—H20*C*⋯*Cg*2^iii^	0.96	2.92	3.740 (3)	144

Symmetry codes: (i) 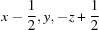; (ii) 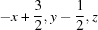; (iii) 
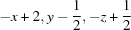
.

**Fig. 1.**
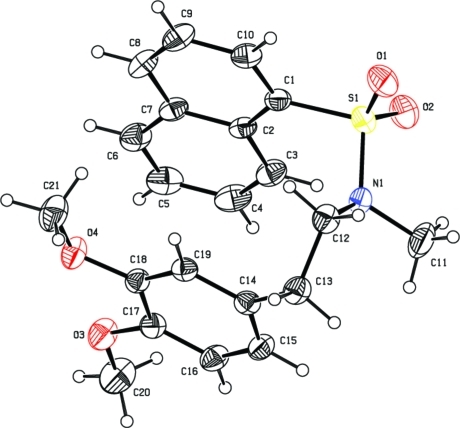
The molecular structure of (I), with atom labels and 30% probability displacement ellipsoids for non-H atoms.

**Fig. 2.**
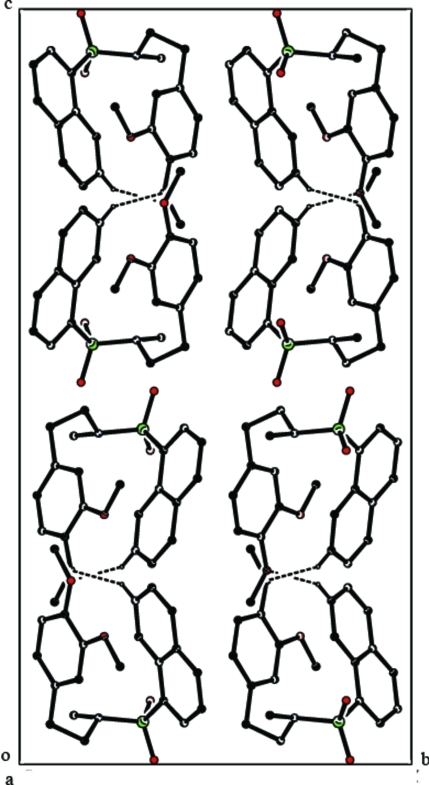
The packing of (I), viewed down the a axis. Intermolecular hydrogen bonds are shown as dashed lines. H atoms not involved in hydrogen bonding have been omitted.

## supplementary crystallographic information

### Comment

In view of the biological activities of sulfonamide derivatives such as insecticidal, antimicrobial and anticancer (Schultz *et al.*, 2001; Sheppard *et al.*, 2006; Xiong *et al.*, 2007), we herewith report the crystal structure of the title compound (I), (Fig. 1). The geometric parameters of the title compound (I) are comparable with the similar reported compounds (Vennila, Thilagavathi *et al.*, 2008; Vennila, Kavitha *et al.*, 2008).

The naphthalene and benzene rings are oriented at an angle of 7.66 (3)°. The molecular structure is stabilized by weak intramolecular C-H···O hydrogen bonds (Table 1) and the crystal structure is formed by weak intermolecular C-H···O (Fig. 2), C-H..π (Table 1) and π–π [Cg1···Cg3 (x,1/2-y,1/2+z) distance 3.710 (2)Å; Cg1 and Cg3 are the centroids of the rings (C1/C2/C7/C8/C9/C10) and (C14-C19), respectively] interactions.

### Experimental

About 5.0 g (25 mmol) of [2-(3, 4-dimethoxy-phenyl)-ethyl]-methyl-amine and 6.8 g (30 mmol) of naphthalene-1-sulfonyl chloride were dissolved in 40mL of methylene dichloride. To this mixture, 5.1 g (51 mmol) of triethylamine was added at 298 K. The reaction mixture was warmed to 313 K and maintained at that temperature for 4 to 6 hrs. The completion of the reaction was checked by TLC. The reaction mixture was cooled to 288 K and added 50 mL of water. The organic layer was separated, washed to neutral pH with 5 % aqueous sodium bicarbonate solution, dried over anhydrous sodium sulphate and concentrated. The crude compound was column purified by using petroleum ether and ethyl acetate. Recrystallisation of the compound with the mixture of ethyl acetate and n-heptane mixture yielded colourless crystals of diffraction quality.

### Refinement

All H atoms were positioned geometrically with C—H = 0.93–0.97 Å and allowed to ride on their parent atoms with *U*_iso_(H) = 1.2Ueq(C) or 1.5Ueq(C_methyl_).

### Crystal data

**Table:**

C_21_H_23_NO_4_S	*F*(000) = 1632
*M**_r_* = 385.46	*D*_x_ = 1.323 Mg m^−^^3^
Orthorhombic, *P**b**c**a*	Mo *K*α radiation, λ = 0.71073 Å
Hall symbol: -P 2ac 2ab	Cell parameters from 26403 reflections
*a* = 10.070 (5) Å	θ = 2.5–29.3°
*b* = 14.120 (4) Å	µ = 0.19 mm^−^^1^
*c* = 27.229 (5) Å	*T* = 295 K
*V* = 3872 (2) Å^3^	Block, colourless
*Z* = 8	0.34 × 0.30 × 0.28 mm

### Data collection

**Table:**

Bruker Kappa APEXII diffractometer	5239 independent reflections
Radiation source: fine-focus sealed tube	3248 reflections with *I* > 2σ(*I*)
Graphite monochromator	*R*_int_ = 0.041
ω and φ scans	θ_max_ = 29.3°, θ_min_ = 2.5°
Absorption correction: multi-scan (*SADABS*; Sheldrick, 1996)	*h* = −13→13
*T*_min_ = 0.937, *T*_max_ = 0.948	*k* = −10→19
9215 measured reflections	*l* = −36→37

### Refinement

**Table:**

Refinement on *F*^2^	Primary atom site location: structure-invariant direct methods
Least-squares matrix: full	Secondary atom site location: difference Fourier map
*R*[*F*^2^ > 2σ(*F*^2^)] = 0.045	Hydrogen site location: inferred from neighbouring sites
*wR*(*F*^2^) = 0.120	H-atom parameters constrained
*S* = 1.01	*w* = 1/[σ^2^(*F*_o_^2^) + (0.0441*P*)^2^ + 1.1352*P*] where *P* = (*F*_o_^2^ + 2*F*_c_^2^)/3
5239 reflections	(Δ/σ)_max_ < 0.001
247 parameters	Δρ_max_ = 0.21 e Å^−^^3^
0 restraints	Δρ_min_ = −0.38 e Å^−^^3^

### Fractional atomic coordinates and isotropic or equivalent isotropic displacement parameters (Å^2^)

**Table:**

	*x*	*y*	*z*	*U*_iso_*/*U*_eq_	
C1	0.92055 (17)	0.36733 (11)	0.08433 (6)	0.0419 (4)	
C2	0.94855 (17)	0.35623 (11)	0.13542 (6)	0.0404 (4)	
C3	0.8706 (2)	0.30395 (13)	0.16901 (7)	0.0524 (5)	
H3	0.7930	0.2749	0.1583	0.063*	
C4	0.9080 (2)	0.29561 (14)	0.21685 (7)	0.0623 (6)	
H4	0.8551	0.2612	0.2384	0.075*	
C5	1.0239 (2)	0.33759 (14)	0.23403 (7)	0.0640 (6)	
H5	1.0485	0.3303	0.2667	0.077*	
C6	1.1009 (2)	0.38904 (14)	0.20327 (7)	0.0583 (5)	
H6	1.1779	0.4173	0.2151	0.070*	
C7	1.06558 (19)	0.40032 (12)	0.15342 (6)	0.0463 (4)	
C8	1.1458 (2)	0.45408 (13)	0.12135 (8)	0.0586 (5)	
H8	1.2222	0.4829	0.1334	0.070*	
C9	1.1141 (2)	0.46457 (14)	0.07347 (8)	0.0614 (5)	
H9	1.1674	0.5012	0.0531	0.074*	
C10	1.0010 (2)	0.42025 (12)	0.05468 (7)	0.0521 (5)	
H10	0.9803	0.4268	0.0216	0.063*	
C11	0.6973 (2)	0.13869 (18)	0.06506 (9)	0.0765 (7)	
H11A	0.6656	0.1242	0.0327	0.115*	
H11B	0.6282	0.1695	0.0834	0.115*	
H11C	0.7224	0.0812	0.0814	0.115*	
C12	0.92293 (19)	0.16608 (13)	0.03129 (6)	0.0498 (4)	
H12A	0.9896	0.2153	0.0282	0.060*	
H12B	0.8901	0.1516	−0.0014	0.060*	
C13	0.98652 (19)	0.07800 (12)	0.05306 (6)	0.0493 (4)	
H13A	0.9214	0.0274	0.0533	0.059*	
H13B	1.0591	0.0583	0.0320	0.059*	
C14	1.03892 (17)	0.09117 (11)	0.10439 (6)	0.0408 (4)	
C15	0.98454 (18)	0.04479 (12)	0.14386 (6)	0.0481 (4)	
H15	0.9130	0.0042	0.1390	0.058*	
C16	1.03471 (19)	0.05758 (12)	0.19086 (6)	0.0496 (4)	
H16	0.9955	0.0264	0.2172	0.059*	
C17	1.14125 (18)	0.11561 (12)	0.19879 (6)	0.0432 (4)	
C18	1.19942 (16)	0.16272 (12)	0.15871 (6)	0.0400 (4)	
C19	1.14727 (16)	0.15094 (12)	0.11246 (6)	0.0396 (4)	
H19	1.1847	0.1833	0.0861	0.048*	
C20	1.1405 (3)	0.08946 (18)	0.28507 (7)	0.0775 (7)	
H20A	1.0503	0.1109	0.2878	0.116*	
H20B	1.1889	0.1075	0.3140	0.116*	
H20C	1.1417	0.0218	0.2818	0.116*	
C21	1.3788 (2)	0.25648 (17)	0.12955 (8)	0.0718 (6)	
H21A	1.4030	0.2076	0.1068	0.108*	
H21B	1.4575	0.2871	0.1415	0.108*	
H21C	1.3233	0.3021	0.1133	0.108*	
N1	0.81255 (14)	0.20151 (10)	0.06160 (5)	0.0460 (4)	
O1	0.78111 (16)	0.34316 (12)	0.00575 (6)	0.0787 (5)	
O2	0.66402 (14)	0.33351 (11)	0.08444 (7)	0.0782 (5)	
O3	1.20031 (14)	0.13109 (10)	0.24318 (4)	0.0611 (4)	
O4	1.30859 (13)	0.21602 (10)	0.16952 (5)	0.0563 (3)	
S1	0.78111 (5)	0.31428 (4)	0.056242 (19)	0.05377 (15)	

### Atomic displacement parameters (Å^2^)

**Table:**

	*U*^11^	*U*^22^	*U*^33^	*U*^12^	*U*^13^	*U*^23^
C1	0.0431 (9)	0.0320 (8)	0.0507 (10)	0.0023 (7)	0.0041 (8)	0.0032 (7)
C2	0.0467 (9)	0.0276 (8)	0.0469 (9)	0.0013 (7)	0.0096 (8)	−0.0001 (7)
C3	0.0557 (11)	0.0467 (11)	0.0549 (11)	−0.0065 (9)	0.0141 (9)	0.0008 (8)
C4	0.0846 (15)	0.0499 (12)	0.0524 (12)	−0.0034 (11)	0.0223 (11)	0.0054 (9)
C5	0.0966 (17)	0.0504 (12)	0.0449 (10)	0.0050 (12)	0.0016 (11)	−0.0040 (9)
C6	0.0765 (14)	0.0458 (11)	0.0526 (11)	−0.0035 (10)	−0.0043 (10)	−0.0114 (9)
C7	0.0569 (11)	0.0311 (9)	0.0510 (10)	−0.0020 (8)	0.0051 (8)	−0.0066 (7)
C8	0.0617 (12)	0.0451 (11)	0.0689 (13)	−0.0199 (9)	0.0048 (10)	−0.0040 (9)
C9	0.0691 (13)	0.0511 (12)	0.0640 (12)	−0.0204 (10)	0.0132 (11)	0.0092 (10)
C10	0.0632 (12)	0.0449 (10)	0.0484 (10)	−0.0020 (9)	0.0045 (9)	0.0100 (8)
C11	0.0538 (13)	0.0750 (16)	0.1008 (18)	−0.0260 (11)	−0.0027 (12)	−0.0060 (13)
C12	0.0542 (11)	0.0564 (11)	0.0389 (9)	−0.0039 (9)	−0.0016 (8)	−0.0026 (8)
C13	0.0562 (11)	0.0451 (10)	0.0467 (10)	−0.0019 (9)	−0.0007 (8)	−0.0122 (8)
C14	0.0465 (9)	0.0346 (8)	0.0413 (9)	0.0060 (7)	0.0008 (7)	−0.0070 (7)
C15	0.0513 (10)	0.0409 (9)	0.0521 (10)	−0.0090 (8)	−0.0017 (9)	−0.0022 (8)
C16	0.0579 (11)	0.0460 (10)	0.0447 (10)	−0.0100 (9)	0.0034 (8)	0.0051 (8)
C17	0.0489 (10)	0.0420 (9)	0.0388 (9)	−0.0004 (8)	−0.0017 (8)	−0.0016 (7)
C18	0.0384 (9)	0.0369 (9)	0.0447 (9)	0.0000 (7)	0.0020 (7)	−0.0030 (7)
C19	0.0416 (9)	0.0367 (9)	0.0405 (9)	0.0037 (7)	0.0068 (7)	−0.0008 (7)
C20	0.1034 (19)	0.0884 (16)	0.0407 (11)	−0.0257 (15)	−0.0018 (12)	0.0064 (11)
C21	0.0554 (12)	0.0881 (17)	0.0717 (14)	−0.0249 (12)	0.0095 (11)	0.0059 (12)
N1	0.0383 (7)	0.0474 (9)	0.0524 (9)	−0.0076 (6)	−0.0042 (6)	0.0007 (7)
O1	0.0816 (11)	0.0849 (11)	0.0697 (10)	−0.0032 (9)	−0.0314 (8)	0.0276 (8)
O2	0.0397 (8)	0.0758 (11)	0.1191 (14)	0.0113 (7)	0.0055 (8)	0.0047 (9)
O3	0.0721 (9)	0.0705 (9)	0.0408 (7)	−0.0192 (7)	−0.0081 (6)	0.0050 (6)
O4	0.0484 (7)	0.0687 (9)	0.0518 (7)	−0.0179 (6)	−0.0003 (6)	0.0008 (6)
S1	0.0413 (2)	0.0552 (3)	0.0648 (3)	0.0029 (2)	−0.0096 (2)	0.0108 (2)

### Geometric parameters (Å, º)

**Table:**

C1—C10	1.366 (2)	C12—H12B	0.9700
C1—C2	1.428 (2)	C13—C14	1.506 (2)
C1—S1	1.7656 (19)	C13—H13A	0.9700
C2—C3	1.413 (2)	C13—H13B	0.9700
C2—C7	1.420 (2)	C14—C15	1.372 (2)
C3—C4	1.361 (3)	C14—C19	1.397 (2)
C3—H3	0.9300	C15—C16	1.388 (2)
C4—C5	1.390 (3)	C15—H15	0.9300
C4—H4	0.9300	C16—C17	1.367 (2)
C5—C6	1.353 (3)	C16—H16	0.9300
C5—H5	0.9300	C17—O3	1.365 (2)
C6—C7	1.412 (3)	C17—C18	1.406 (2)
C6—H6	0.9300	C18—O4	1.364 (2)
C7—C8	1.411 (3)	C18—C19	1.375 (2)
C8—C9	1.350 (3)	C19—H19	0.9300
C8—H8	0.9300	C20—O3	1.418 (2)
C9—C10	1.396 (3)	C20—H20A	0.9600
C9—H9	0.9300	C20—H20B	0.9600
C10—H10	0.9300	C20—H20C	0.9600
C11—N1	1.464 (2)	C21—O4	1.418 (2)
C11—H11A	0.9600	C21—H21A	0.9600
C11—H11B	0.9600	C21—H21B	0.9600
C11—H11C	0.9600	C21—H21C	0.9600
C12—N1	1.472 (2)	N1—S1	1.6300 (16)
C12—C13	1.519 (2)	O1—S1	1.4341 (15)
C12—H12A	0.9700	O2—S1	1.4330 (16)
			
C10—C1—C2	121.23 (16)	C14—C13—H13B	108.7
C10—C1—S1	116.60 (14)	C12—C13—H13B	108.7
C2—C1—S1	122.17 (12)	H13A—C13—H13B	107.6
C3—C2—C7	117.79 (17)	C15—C14—C19	118.48 (16)
C3—C2—C1	125.33 (17)	C15—C14—C13	121.88 (16)
C7—C2—C1	116.87 (15)	C19—C14—C13	119.63 (15)
C4—C3—C2	120.74 (19)	C14—C15—C16	120.98 (17)
C4—C3—H3	119.6	C14—C15—H15	119.5
C2—C3—H3	119.6	C16—C15—H15	119.5
C3—C4—C5	121.16 (19)	C17—C16—C15	120.63 (16)
C3—C4—H4	119.4	C17—C16—H16	119.7
C5—C4—H4	119.4	C15—C16—H16	119.7
C6—C5—C4	120.11 (19)	O3—C17—C16	125.31 (16)
C6—C5—H5	119.9	O3—C17—C18	115.48 (16)
C4—C5—H5	119.9	C16—C17—C18	119.20 (16)
C5—C6—C7	120.8 (2)	O4—C18—C19	124.91 (15)
C5—C6—H6	119.6	O4—C18—C17	115.41 (15)
C7—C6—H6	119.6	C19—C18—C17	119.66 (16)
C8—C7—C6	120.76 (18)	C18—C19—C14	121.04 (15)
C8—C7—C2	119.83 (17)	C18—C19—H19	119.5
C6—C7—C2	119.41 (17)	C14—C19—H19	119.5
C9—C8—C7	121.38 (19)	O3—C20—H20A	109.5
C9—C8—H8	119.3	O3—C20—H20B	109.5
C7—C8—H8	119.3	H20A—C20—H20B	109.5
C8—C9—C10	119.84 (18)	O3—C20—H20C	109.5
C8—C9—H9	120.1	H20A—C20—H20C	109.5
C10—C9—H9	120.1	H20B—C20—H20C	109.5
C1—C10—C9	120.81 (18)	O4—C21—H21A	109.5
C1—C10—H10	119.6	O4—C21—H21B	109.5
C9—C10—H10	119.6	H21A—C21—H21B	109.5
N1—C11—H11A	109.5	O4—C21—H21C	109.5
N1—C11—H11B	109.5	H21A—C21—H21C	109.5
H11A—C11—H11B	109.5	H21B—C21—H21C	109.5
N1—C11—H11C	109.5	C11—N1—C12	115.39 (16)
H11A—C11—H11C	109.5	C11—N1—S1	116.34 (14)
H11B—C11—H11C	109.5	C12—N1—S1	115.37 (12)
N1—C12—C13	112.19 (14)	C17—O3—C20	117.45 (16)
N1—C12—H12A	109.2	C18—O4—C21	117.28 (15)
C13—C12—H12A	109.2	O2—S1—O1	117.38 (10)
N1—C12—H12B	109.2	O2—S1—N1	107.27 (9)
C13—C12—H12B	109.2	O1—S1—N1	111.32 (9)
H12A—C12—H12B	107.9	O2—S1—C1	109.99 (10)
C14—C13—C12	114.14 (14)	O1—S1—C1	107.14 (9)
C14—C13—H13A	108.7	N1—S1—C1	102.78 (8)
C12—C13—H13A	108.7		
			
C10—C1—C2—C3	179.26 (17)	C15—C16—C17—C18	0.3 (3)
S1—C1—C2—C3	−0.5 (2)	O3—C17—C18—O4	1.2 (2)
C10—C1—C2—C7	−1.8 (2)	C16—C17—C18—O4	−177.34 (16)
S1—C1—C2—C7	178.39 (12)	O3—C17—C18—C19	179.58 (15)
C7—C2—C3—C4	−0.7 (3)	C16—C17—C18—C19	1.0 (3)
C1—C2—C3—C4	178.20 (17)	O4—C18—C19—C14	176.77 (15)
C2—C3—C4—C5	−0.4 (3)	C17—C18—C19—C14	−1.4 (2)
C3—C4—C5—C6	1.0 (3)	C15—C14—C19—C18	0.5 (2)
C4—C5—C6—C7	−0.5 (3)	C13—C14—C19—C18	−178.45 (15)
C5—C6—C7—C8	179.94 (19)	C13—C12—N1—C11	−65.3 (2)
C5—C6—C7—C2	−0.6 (3)	C13—C12—N1—S1	154.41 (12)
C3—C2—C7—C8	−179.36 (17)	C16—C17—O3—C20	−5.1 (3)
C1—C2—C7—C8	1.6 (2)	C18—C17—O3—C20	176.43 (18)
C3—C2—C7—C6	1.2 (2)	C19—C18—O4—C21	−4.9 (3)
C1—C2—C7—C6	−177.81 (16)	C17—C18—O4—C21	173.40 (17)
C6—C7—C8—C9	179.26 (19)	C11—N1—S1—O2	34.67 (17)
C2—C7—C8—C9	−0.2 (3)	C12—N1—S1—O2	174.52 (13)
C7—C8—C9—C10	−1.2 (3)	C11—N1—S1—O1	−95.00 (16)
C2—C1—C10—C9	0.5 (3)	C12—N1—S1—O1	44.86 (15)
S1—C1—C10—C9	−179.67 (15)	C11—N1—S1—C1	150.62 (15)
C8—C9—C10—C1	1.0 (3)	C12—N1—S1—C1	−69.52 (14)
N1—C12—C13—C14	−57.6 (2)	C10—C1—S1—O2	−130.70 (15)
C12—C13—C14—C15	113.75 (19)	C2—C1—S1—O2	49.10 (16)
C12—C13—C14—C19	−67.3 (2)	C10—C1—S1—O1	−2.07 (17)
C19—C14—C15—C16	0.7 (3)	C2—C1—S1—O1	177.73 (14)
C13—C14—C15—C16	179.70 (16)	C10—C1—S1—N1	115.32 (14)
C14—C15—C16—C17	−1.1 (3)	C2—C1—S1—N1	−64.88 (15)
C15—C16—C17—O3	−178.17 (17)		

### Hydrogen-bond geometry (Å, º)

*Cg*2 and *Cg*3 are the centroids of the C2–C7 and C14–C19 rings, respectively.

**Table:**

*D*—H···*A*	*D*—H	H···*A*	*D*···*A*	*D*—H···*A*
C3—H3···O2	0.93	2.53	3.132 (3)	122
C10—H10···O1	0.93	2.37	2.804 (3)	109
C11—H11*B*···O2	0.96	2.34	2.821 (3)	110
C4—H4···O3^i^	0.93	2.46	3.310 (3)	152
C8—H8···*Cg*3^ii^	0.93	2.59	3.488 (3)	163
C20—H20*C*···*Cg*2^iii^	0.96	2.92	3.740 (3)	144

Symmetry codes: (i) *x*−1/2, *y*, −*z*+1/2; (ii) −*x*+3/2, *y*−1/2, *z*; (iii) −*x*+2, *y*−1/2, −*z*+1/2.
